# A High-PSRR LDO with Low Noise and Ultra-Low Power Consumption

**DOI:** 10.3390/mi17010091

**Published:** 2026-01-10

**Authors:** Nanxiang Guo, Jiagen Cheng, Chenxi Yue, Changtao Chen, Chaoran Liu, Linxi Dong

**Affiliations:** 1 Department of Electrical and Electronic Engineering, Luzhou Vocational & Technical College, Luzhou 646100, China; gnx1980@163.com (N.G.); cct001@163.com (C.C.); 2 Luzhou Key Laboratory of Intelligent Control and Applications Technology of Electronic Devices, Luzhou 646100, China; 3 College of Electronics and Information, Hangzhou Dianzi University, Hangzhou 310018, China; cjg19960325@163.com (J.C.); 202241040008@hdu.edu.cn (C.Y.); liucr@hdu.edu.cn (C.L.)

**Keywords:** low dropout regulator, high power supply rejection ratio, low noise, low standby current

## Abstract

High-performance low dropout regulator (LDO) chips are core components that provide clean power for high-precision sensors, radio frequency (RF) circuits, low noise amplifiers and other noise-sensitive circuits. In the reported literature, the designed LDO chip has advantages in certain parameters, but it cannot meet all the requirements of a high power supply rejection ratio (PSRR), low output noise and low standby current at the same time, which makes the high-end applications of LDOs greatly limited. In this paper, an LDO chip with high PSRR, low output noise and low standby current has been designed and fabricated. By increasing the loop gain, introducing an improved feedforward path, and adopting isolated power supply, the PSRR of the LDO at different frequency bands is greatly improved. By optimizing the design of the error amplifier (EA) and adding a low-pass filter to filter out the reference noise, the output voltage noise of the LDO is reduced. Within the depletion process and an optimized reference structure, the standby power consumption of the LDO is reduced without damaging the output voltage accuracy. The chip is taped out with SMIC’s 0.18 μm/5 V/BCD process. The measured PSRR of the chip is as high as 95dB at a frequency of 1 kHz, and the high-frequency (1 MHz) PSRR is above 45 dB. The amplitude of integrated output noise is below 5.4 μVrms within the frequency range of 10 Hz to 100 KHz. When the load current is zero, the measured standby current is less than 400 nA. The test results indicate that the chip has excellent performance in terms of PSRR, output noise and standby power consumption.

## 1. Introduction

In the power management system, high-performance LDOs are widely used, especially in aerospace, 5G communication, weak analog signal detection and other applications that are very sensitive to power noise. An LDO with high PSRR can effectively filter the voltage ripple generated by the upper power stage, usually composed of switching convertors. The internal circuit of an LDO should not introduce additional noise into the output voltage, so the thermal noise and 1/f noise of the internal devices must be suppressed [1]. With the popularity of solar panels and lithium batteries as power supplies, to extend system runtime, ultra-low standby power consumption is required [2].

A common way to improve the PSRR is to use an N-type metal oxide semiconductor (NMOS) as the power MOSFET [3]. However, the drive voltage of NMOS needs to be raised by a charge pump, which makes the circuit design more complex and makes it difficult to achieve ultra-low power dissipation. In [4], a multi-stage voltage stabilization technology is adopted, and the PSRR of the LDO can reach 110 dB at 1 kHz, but this design does not take the power consumption into consideration. Mohamed El-Nozahi performed a detailed analysis of LDOs’ PSRR [5]. According to his work, the value of PSRR depends on the open-loop gain of the LDO. However, with the increase in operating frequency, the loop gain will gradually decline due to the parasitic capacitance, resulting in serious attenuation of PSRR at medium and high frequencies. Bangda Yang proposed a supply-noise cancellation technology by adding a feedforward path into the loop to effectively improve the high-frequency characteristics of PSRR [6]. However, the implementation of this method still consumes a lot of current and cannot achieve low standby current consumption. The integrated output noise of LDOs is mainly generated by the thermal noise and 1/f noise of internal devices. The most effective way to suppress device noise is to increase the bias current, which will also simultaneously increase the standby current.

In this paper, a two-stage operational amplifier is used to increase the open-loop gain to improve the DC-PSRR of the LDO chip. By inserting a coupling capacitor between a critical node of the operational amplifier and the power line, a feedforward path is formed, which introduces a pole-zero doublet, thus improving the high-frequency PSRR of the LDO. Since the reference voltage will fluctuate due to power supply ripple, and the fluctuation will be transmitted to the output through the error amplifier (EA), the PSRR of the reference voltage source needs to be improved. In [7], the power supply ripple is directly filtered out by a low-pass filter, which will seriously degrade the dynamic characteristics. In this paper, a low-pass filter is inserted between the reference voltage and the EA input, which can filter out the power fluctuation and the reference noise simultaneously. The output noise of the LDO mainly comes from the EA and the reference voltage source [8]. The power transistor contributes very little noise to the system due to its large area and its location at the system end. To reduce the noise of the EA, chopping technology is adopted [9]. However, this will greatly increase the power consumption of the chip. J. Mihhailov provided a detailed analysis of LDO noise at the system level [10]. The noise of the reference voltage is difficult to suppress by simply optimizing the circuit structure. In this paper, by optimizing EA design and inserting a low-pass filter between the reference and the EA’s negative input port, low output noise is achieved. In order to reduce the standby power consumption, a depletion process is adopted and the depletion N-type transistor is used in the design of the reference voltage source. The traditional depletion reference voltage source is realized by connecting the depletion and enhancement transistors in series [11,12], and the consumed current can be as low as 20 nA or less. The greatest disadvantage of the traditional structure is that the drift of the reference voltage is large and difficult to trim using fuses. To address this problem, the circuit is reconstructed and the reference voltage is generated by a current with zero temperature coefficient flowing through the resistor. In this paper, the characteristics of PSRR and output voltage noise are first analyzed, and then the key circuit is designed. Finally, the test results of the designed LDO chip are given.

## 2. Analysis of PSRR and Construction of Output Noise Model

### 2.1. Analysis of LDO’s PSRR

**Figure 1:**
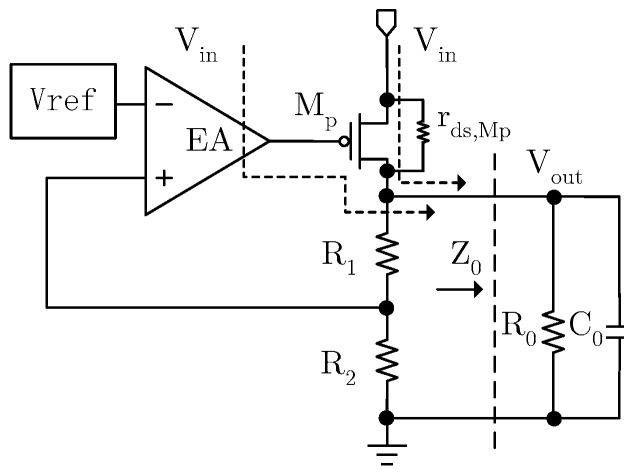
Path of PSRR.

As shown in Figure 1, the power supply influences the output voltage of LDOs mainly through the power transistor, the EA and the reference voltage source. As the reference voltage is stabilized by the low-pass filter, the impact of the reference voltage on the PSRR of the system can be ignored. Firstly, the influence of the power supply *V_in_* on the output voltage through the power transistor *M_p_* is calculated. The output voltage variation will be fed back to the positive input port of the EA with the feedback ratio determined by the voltage divider resistors, and will be applied to the gate of *M_p_* after being amplified by the EA. Based on the current balance at the output port, the following can be obtained:

(1) $$g_{Mp}(\Delta V_{in}-A_{ea}\frac{R_{2}}{R_{1}+R_{2}}\Delta V_{out})+\frac{\Delta V_{in}-\Delta V_{out}}{r_{ds,Mp}}=\frac{\Delta V_{out}}{Z_{0}}$$

Δ*V_out_* represents the variation in the output voltage, *g_Mp_* is the transconductance of the power transistor, *A_ea_* represents the gain of the EA, *r_ds,Mp_* represents the channel resistance of the power transistor and *Z*_0_ is the equivalent impedance at the output terminal. Due to their large values, *R*_1_ and *R*_2_ can be ignored in the calculation of *Z*_0_ to simplify the analysis. The relationship between the power supply variation and the output voltage can be expressed as

(2) $$\frac{\Delta V_{out}}{\Delta V_{in}}=\frac{g_{Mp}Z_{0}r_{ds,Mp}+Z_{0}}{Z_{0}+r_{ds,Mp}+g_{Mp}A_{ea}\frac{R_{2}}{R_{1}+R_{2}}Z_{0}r_{ds,Mp}}$$

Equation (2) is the expression for the *PSRR* of the LDO that considers only the influence of the power transistor. It can be seen from Equation (2) that as the operation frequency is very low, the *PSRR* is determined entirely by the gain of the EA. However, as the frequency increases, the gain of the EA decreases due to parasitic capacitance, and the *PSRR* of the LDO will continue to deteriorate. Secondly, considering the path from the EA, by equating the impact of power supply variation on the EA output to that at the EA input port, the following expression can be derived:

(3) $$\Delta V_{in,ea}=\Delta V_{in}PSRR_{ea}$$

Δ*V_in,ea_* represents the input voltage change of the EA caused by power supply variation, and *PSRR_ea_* is the *PSRR* of the EA. Assuming that the output voltage change of the LDO is Δ*V_out_*, the closed-loop gain can be calculated:

(4) $$\Delta V_{out}=(\Delta V_{in}PSRR_{ea}-\frac{R_{2}}{R_{1}+R_{2}}\Delta V_{out})A_{ea}g_{Mp}Z_{0}$$

Simplifying Equation (4), the following can be written:

(5) $$\frac{\Delta V_{out}}{\Delta V_{in}}=\frac{g_{Mp}Z_{0}A_{ea}PSRR_{ea}}{1+\frac{R_{2}}{R_{1}+R_{2}}g_{Mp}Z_{0}A_{ea}}$$

Equation (5) indicates that when suppressing the influence of power supply variation on the LDO’s output voltage through the EA, the most effective way is to improve the *PSRR* of the EA. In summary, it can be concluded that to improve the *PSRR* of LDO, the DC gain of the EA should exceed the target value of the system’s *PSRR*, and its *PSRR* should be maximized.

### 2.2. Model of LDO Output Voltage Noise

**Figure 2:**
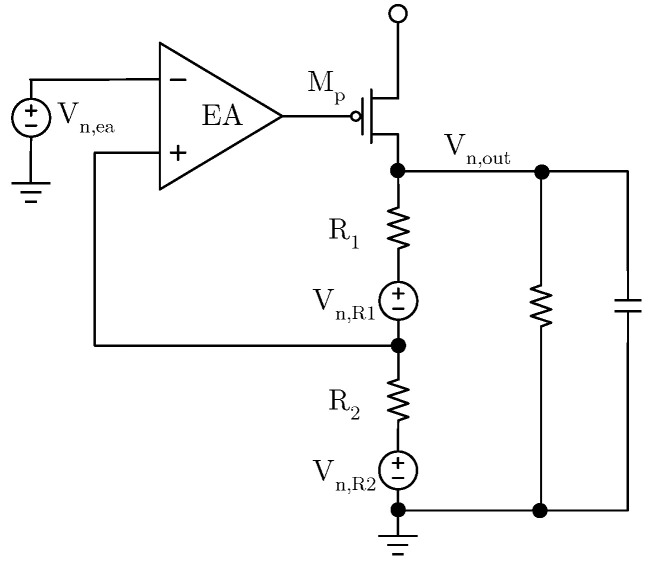
Main noise source of LDO.

Due to the difficulty in suppressing the noise of the reference voltage, which can only be filtered out by a low-pass filter [13,14] and the negligible 1/f noise of the power transistor, the contribution of output voltage noise mainly comes from the EA and feedback resistors. As shown in Figure 2, *V_n,ea_* is the equivalent input noise of the EA, *V_n,R_*_1_ and *V_n,R_*_2_ represent the thermal noise of *R*_1_ and *R*_2_, respectively, and *V_n,out_* represents the noise at the output of the LDO. Since the noise has no direction, the sign of the noise is not considered. It can be concluded that

(6) $$V_{n,out}=(V_{n,ea}+V_{n,R_{1}}\frac{R_{2}}{R_{1}}+V_{n,R_{2}}\frac{R_{1}}{R_{1}+R_{2}})\frac{A_{ea}}{1+\frac{R_{1}}{R_{1}+R_{2}}A_{ea}}$$

Equation (6) can be simplified:

(7) $$V_{n,out}=V_{n,ea}\frac{R_{1}+R_{2}}{R_{2}}+V_{n,R_{1}}+V_{n,R_{2}}\frac{R_{1}}{R_{2}}$$

**Figure 3:**
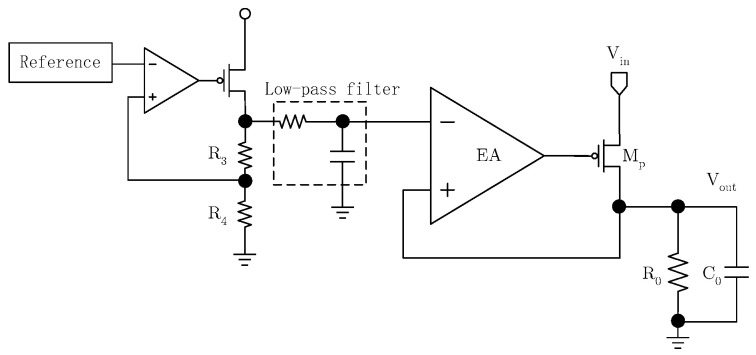
Circuit scheme of LDO.

According to Equation (7), when R1 is zero, the output voltage noise is minimized. The equivalent input noise of the EA equals the LDO’s output voltage noise. In practice, it is impossible to completely eliminate the reference voltage noise. Consequently, the LDO’s output noise is mainly composed of the filtered reference voltage noise and the EA’s equivalent input noise. The overall LDO circuit schematic is shown in Figure 3. Since the output voltage is directly fed back to the EA’s positive input port, the reference voltage must first be pre-amplified and low-pass-filtered before it is applied to the EA’s negative input terminal. Since the 1/f noise of MOS transistors increases at a lower frequency, and the output noise within the frequency range of 10 Hz to 100 KHz is mainly concerned, the 3 dB bandwidth of the low-pass filter is designed to be around 1 Hz to 2 Hz. Moreover, during startup, the filter capacitor needs to be quickly pre-charged to avoid an excessively long startup time.

## 3. Design of the Key Circuit

### 3.1. Design of EA and Feedforward Path

**Figure 4:**
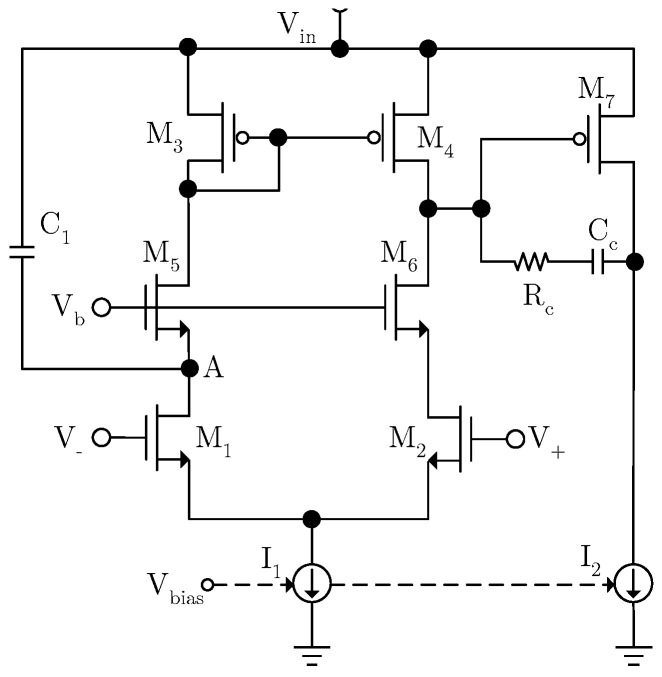
Internal circuit of EA.

The design of the EA is especially crucial because its DC gain determines the upper limit of the PSRR of the LDO. A fast feedforward path is introduced by adding coupling capacitors between the power supply and the EA’s internal node [15,16]. The equivalent input noise of the EA is directly reflected at the output node. Furthermore, the system’s stability must be considered when designing the EA. The circuit is shown in Figure 4.

**Figure 5:**
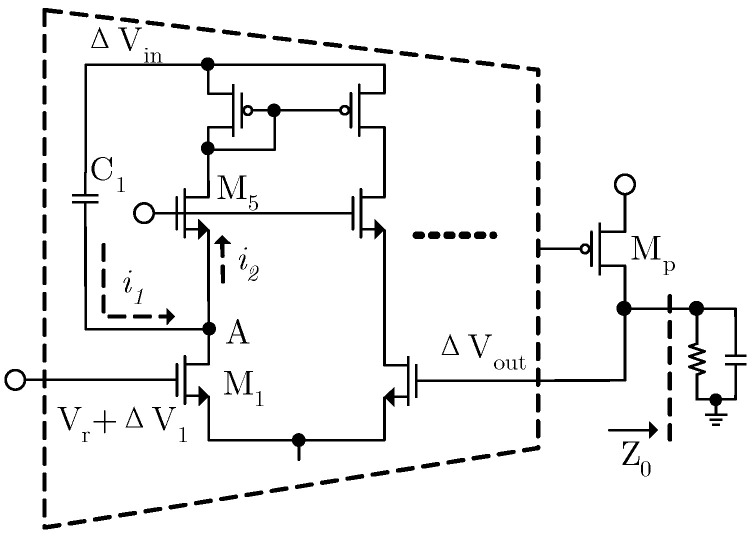
Operating principle of coupling capacitor.

In order to improve DC gain, a two-stage operational amplifier is adopted, achieving a gain of 95 dB. M_5_ and M_6_ are used to suppress the influence of power supply ripple at the EA’s input and enhance the PSRR of the EA. P-type transistors are not used for power supply isolation, mainly because they will lead to excessive loop gain, making compensating the loop difficult. Additionally, due to the lower dominant pole, the transient response of the LDO cannot meet the requirements. The bias voltage V_b_ is a fixed voltage. Since the EA’s output directly drives the power transistor, its large parasitic capacitance creates a low-frequency pole at the EA’s output. To ensure system stability, R_c_ and C_c_ form Miller compensation, which pushes the EA’s output pole to a higher frequency. The dominant pole of the system is formed at the first stage of the EA. Since the zero introduced by Miller compensation cancels the LDO’s output pole, the maximum phase shift remains below 180 degrees, ensuring system stability. As the EA’s gain decreases with increasing frequency, the LDO’s high-frequency PSRR performance degrades. To address this, a coupling capacitor is inserted between the power supply and node A to improve the LDO’s high-frequency PSRR. The operating principle is illustrated in Figure 5.

Assuming that the variation in the power supply is Δ*V_in_* and the equivalent impedance seen from A is about 1/*g_m_*_5_, then the high-frequency coupling current is

(8) $$i_{1}=\frac{sC_{1}g_{m5}\Delta V_{in}}{g_{m5}+sC_{1}}$$

*g_m_*_5_ is the transconductance of M_5_. The equivalent impedance seen from the source of M_5_ is quite small, and then the majority of the coupling current will flow into the current mirror from the source of M_5_ and influence the loop characteristics. For convenience of calculation, the coupling current is converted into the input voltage of the EA. This gives

(9) $$\Delta V_{1}=\frac{sC_{1}g_{m5}\Delta V_{in}}{g_{m1}(g_{m5}+sC_{1})}$$

*g_m_*_1_ is the transconductance of M_1_, and the coupling current is equivalent to the negative input of the EA. This gives

(10) $$(\Delta V_{out}-\Delta V_{1})A_{ea}g_{mp}Z_{0}=\Delta V_{out}$$

By substituting Equation (10) into Equation (9), this gives

(11) $$\frac{\Delta V_{out}}{\Delta V_{in}}=\frac{sC_{1}g_{m5}}{g_{m1}(g_{m5}+sC_{1})}\cdot \frac{A_{ea}g_{mp}Z_{0}}{\left(A_{ea}g_{mp}Z_{0}-1\right)}$$

**Figure 6:**
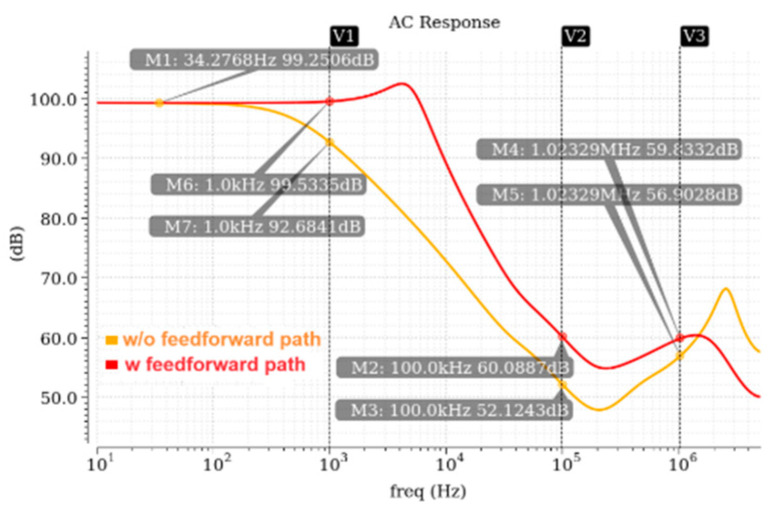
PSRR simulation wave with or without feedforward path.

Equation (11) shows that the coupling capacitor introduces a pole-zero doublet into the PSRR transfer function, which will delay the decay rate of PSRR and improve the PSRR of the LDO in the middle–high-frequency band. Figure 6 shows the simulation comparison of PSRR with and without the improved feedforward path. The pole-zero doublet introduced by the feedforward path increases the dominant pole of the PSRR transfer function to around 5 kHz, improving the PSRR by approximately 7 dB at 1 kHz, 8 dB at 100 kHz, and 3 dB at 1 MHz. The overall PSRR performance is significantly improved.

### 3.2. Design of Depletion Reference Voltage Source

**Figure 7:**
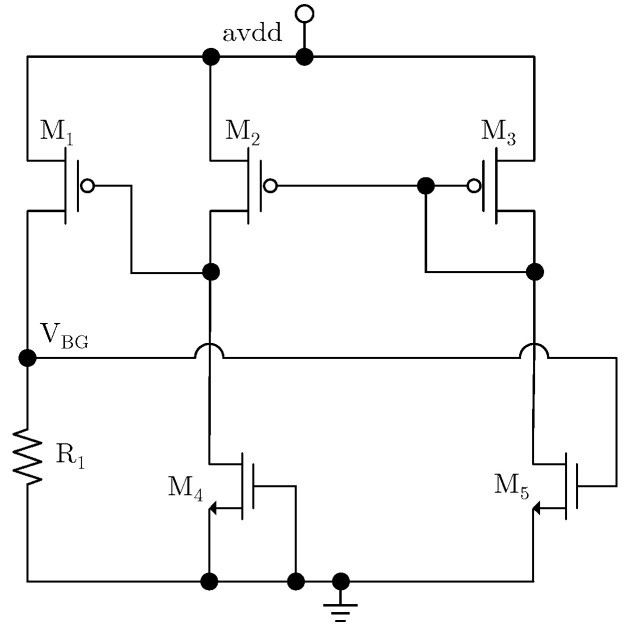
Circuit of depletion reference voltage source.

To reduce the standby power consumption of the chip, the reference voltage source is implemented with depletion technology. The voltage drift of the traditional depletion reference voltage source is usually large and difficult to trim back due to its series structure [17,18]. Azimi M. presents a subthreshold reference with an improved temperature coefficient through body bias curvature compensation [19]. However, this structure exhibits poor rejection of supply noise, resulting in low system PSRR. In this paper, a current mirror is adopted to mirror the current with a zero temperature coefficient onto a resistor to generate the reference voltage, which makes the reference voltage easy to trim and provides better rejection of power supply variations. The circuit is shown in Figure 7.

As Figure 7 shows, M_4_ is a depletion transistor. The *W*/*L* ratios of M_1_, M_2_ and M_3_ are N:1:1, therefore, the current flowing through M_4_ and M_5_ is the same. Assuming a current I, this gives

(12) $$I=\frac{1}{2}\mu_{n}C_{ox}{\left(\frac{W}{L}\right)}_{4}{\left(0-V_{th4}\right)}^{2}=\frac{1}{2}\mu_{n}C_{ox}{\left(\frac{W}{L}\right)}_{5}{\left(V_{BG}-V_{th5}\right)}^{2}$$

*V_th_*_4_ and *V_th_*_5_ represent the threshold voltages of M_4_ and M_5_, respectively, while (*W*/*L*)_4_ and (*W*/*L*)_5_ are the aspect ratios of M_4_ and M_5_. Simplifying Equation (9) yields the following expression:

(13) $$V_{BG}=\sqrt{{\left(\frac{W}{L}\right)}_{4}/{\left(\frac{W}{L}\right)}_{5}}\left|V_{th4}\right|+\left|V_{th5}\right|$$

Since the threshold voltage of depletion-mode MOS transistors is negative, their temperature coefficient is opposite to that of enhancement-mode MOS transistors. Therefore, by adjusting the *W*/*L* ratios of M_4_ and M_5_, a reference voltage with zero temperature coefficient can be obtained. As shown in Figure 7, this gives

(14) $$V_{BG}=N\cdot I\cdot R_{1}$$

**Figure 8:**
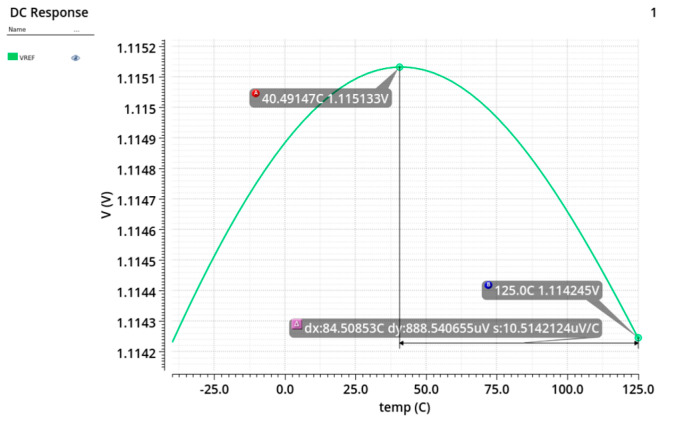
Temperature DC sweep simulation of the reference voltage: reference voltage vs. temperature.

*N* is the current mirror ratio of M_1_. From Equation (14), the reference voltage can be adjusted by trimming the resistance of *R*_1_. Compared with the traditional depletion-mode reference voltage source, the circuit shown in Figure 7 significantly simplifies reference voltage trimming. To improve the PSRR, the depletion-mode reference voltage source is powered by a source-follower, which provides enhanced isolation from the power supply. Figure 8 shows the temperature DC sweep simulation result of the reference voltage. The average temperature coefficient (TC) of the simulated reference voltage is 9.48 ppm/°C.

## 4. Test Results

**Figure 9:**
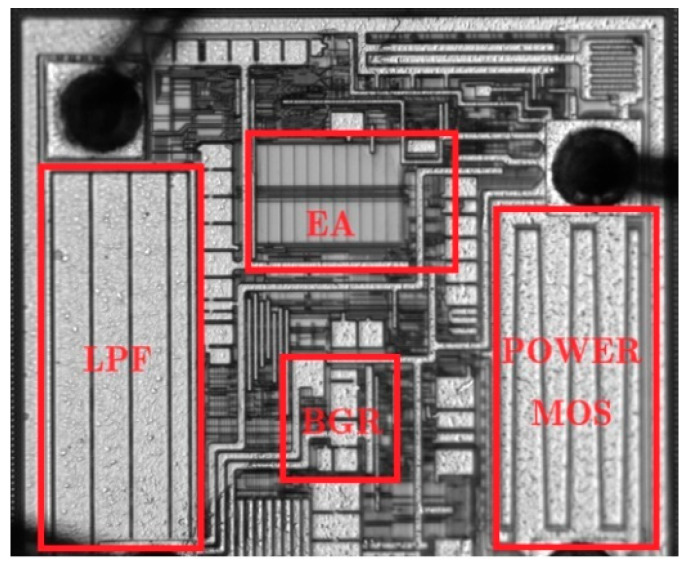
Physical photo of LDO chip.

**Figure 10:**
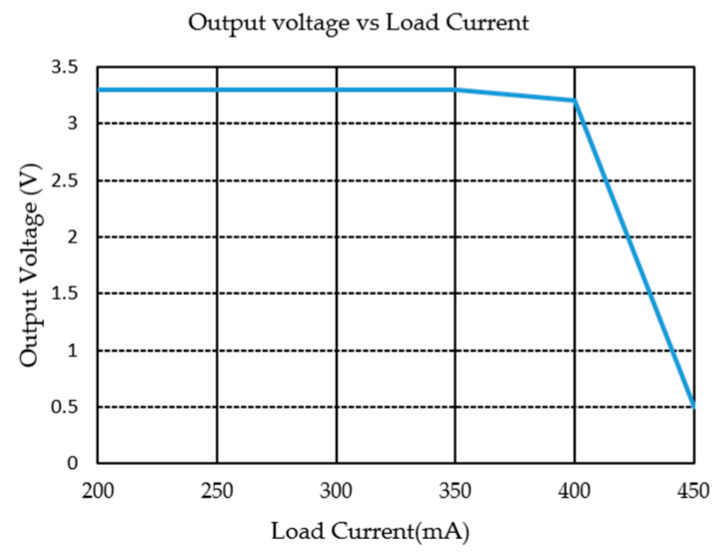
The output voltage vs. load current.

**Figure 11:**
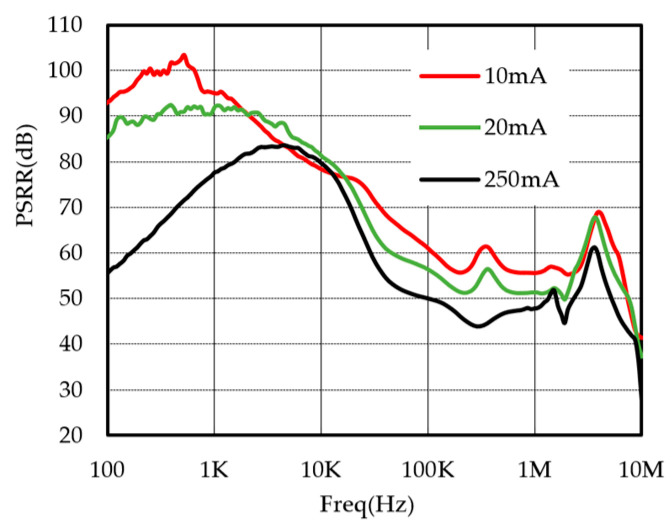
The measured PSRR of the chip.

The LDO chip designed in this paper is fabricated with the SMIC 0.18-μm/5 V BCD process, and the total chip area is 0.27 mm^2^ (520 μm × 520 μm), as shown in Figure 9. The output voltage of the LDO is 3.3 V. The PSRR of the LDO was measured under varying load conditions with an input voltage of 4.3 V. Figure 10 shows the measurement results of the output voltage vs. load current. With an output voltage of 3.3 V and an output current limit of approximately 400 mA, the LDO can provide a sufficient current of 350 mA, meeting the requirements of the vast majority of applications. The PSRR test results are shown in Figure 11. When the load current is 10 mA, the PSRR at 1 kHz reaches 95 dB. By using of the feedforward path, the chip maintains a PSRR greater than 45 dB across the frequency from 100 kHz to 1 MHz. In contrast, the PSRR of other LDO products, such as the TPS7A21, drops below 20 dB within the same frequency band.

**Figure 12:**
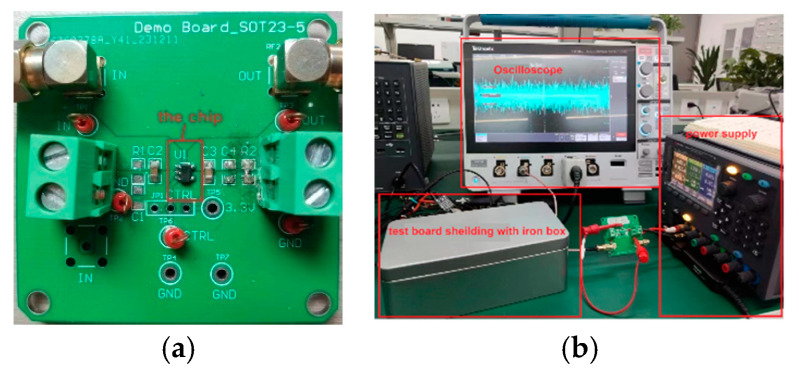
(**a**) Demo of the chip; (**b**) test bench of LDO output noise.

**Figure 13:**
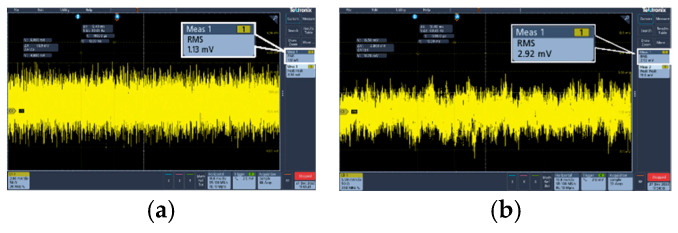
(**a**) Wave of oscilloscope and operational amplifier noise; (**b**) overall LDO output noise.

The amplitude of the output voltage noise is too small to be measured directly, so it needs to be amplified first, and the test board should be shielded with a metal box to avoid external noise interference [20]. The noise testing platform is shown in Figure 12, and the test results of LDO output voltage noise are shown in Figure 13. The overall amplified noise is 2.92 mV_rms_. Both the operational amplifier used for noise amplification and the oscilloscope contain a certain amount of noise, which needs to be removed. As shown in Figure 13, the measured noise of the oscilloscope and operational amplifier is 1.13 mV_rms_. Therefore, the calculated LDO output noise is 2.692 mV_rms_. When divided by an amplification factor of 500, the actual output voltage noise of the LDO is 5.384 μV_rms_.

**Figure 14:**
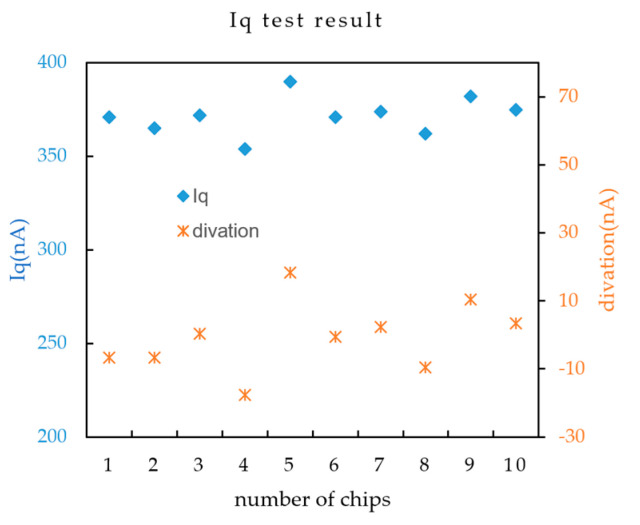
Standby current test result.

When the output load is zero, the current flowing from the ground pin is the standby current of the chip. A 100 Ω resistor connects the chip ground and the test board ground. The standby current is calculated by measuring the voltage drop across the resistor, which must be amplified with a low-offset, low-noise operational amplifier because the standby current is too small. The actual test results are shown in Figure 14, and the standby current of the chip is less than 400 nA, with a maximum deviation of less than 20 nA.

Table 1 summarizes the performance comparison of the proposed LDOs. Compared with previous work, the LDO presented in this work exhibits significant advantages in standby power consumption while maintaining sufficient load capacity. Its PSRR and output noise performance are slightly lower than those of the LDO reported in reference [4], but the design in reference [4] generates a significantly higher quiescent current.

## 5. Conclusions

This paper presents a high-PSRR, low-noise, low-standby-current LDO chip. The measured PSRR of the chip reaches 95 dB. The output voltage noise is less than 6 μVrms, and the standby current is below 400 nA. The test results demonstrate that the chip has been successfully fabricated. A simplified feedforward path is designed and verified, which significantly improves the PSRR of the LDO in the mid-to-high frequency range. Additionally, a unique depletion-type reference voltage source is proposed, enabling easy trimming of the reference voltage and promoting the industrial application of high-performance LDO chips.

## Figures and Tables

**Table 1:** Performance summary of proposed LDO and comparison.

Measured Electric Parameters	Paper [3]	Paper [4]	TPS7A21	This Work
Output current (mA) Test condition: V_in_ = V_out_ + 1V	50	800	500	350
PSRR@1KHz (dB) Test condition: V_in_ = V_out_ + 1V, I_out_ = 10 mA	90	113	91	95
Quiescent current (μA) Test condition: I_out_ = 0	365	>4300	6.5	0.4
Output noise voltage (μV_rms_) BW = 10 Hz to 100 KHz, I_out_ = 1 mA	6.77	5.3	7.7	5.5

## Data Availability

The original contributions presented in this study are included in the article. Further inquiries can be directed to the corresponding author.

## Abbreviations

The following abbreviations are used in this manuscript:

PSRR	Power supply rejection ratio
LDO	Low dropout regulator
RF	Radio frequency
EA	Error amplifier
NMOS	N-type metal oxide semiconductor
